# Primary Nonseminomatous Germ Cell Tumor of Kidney: An Uncommon Renal Neoplasm

**DOI:** 10.15586/jkcvhl.v11i4.335

**Published:** 2024-10-01

**Authors:** Sonu Plash, Deepti Soni, Sramana Mukhopadhyay, Moorat Singh Yadav, Devashish Kaushal, Ketan Mehra

**Affiliations:** 1 Department of Urology, All India Institute of Medical Sciences (AIIMS) Bhopal, Saket Nagar, Habib Ganj, Bhopal, Madhya Pradesh 462026, India;; 2 Department of Pathology, All India Institute of Medical Sciences (AIIMS) Bhopal, Saket Nagar, Habib Ganj, Bhopal, Madhya Pradesh 462026, India;; 3 Department of Surgery, All India Institute of Medical Sciences (AIIMS) Bhopal, Saket Nagar, Habib Ganj, Bhopal, Madhya Pradesh 462026, India

**Keywords:** extragonadal germ cell tumor, germ cell tumor, kidney tumor, nonseminomatous tumor, renal neoplasm

## Abstract

Germ cell tumor (GCT) is a neoplasm typically found in childhood, commonly originating from the testis or ovary. While there have been reported cases of GCT occurring in various extragonadal sites, primary intrarenal GCT is exceptionally rare. We present a case of 37-year-old male who presented with right upper abdomen pain. Imaging revealed a sizable mass within the right kidney. The patient underwent surgical resection of the renal mass during which there was perirenal infiltration into the duodenum and dense desmoplastic reaction all around. Subsequent histopathology confirmed the diagnosis of primary intrarenal nonseminomatous germ cell tumor (NSGCT). The patient underwent four cycles of adjuvant bleomycin, etoposide, and cisplatin (BEP) chemotherapy; at 6 months of follow-up, he is fine. The objective of this case report is to underscore the importance of considering NSGCT as a potential rare differential diagnosis in cases of renal neoplasms and further plan for the management.

## Introduction

Malignant nonseminomatous germ cell tumor (NSGCT) typically originates in the gonads of children. Extragonadal germ cell tumors typically arise in midline locations in both children and adults, particularly in the retroperitoneum, anterior mediastinum, sacrococcygeal region, and pineal gland (1). The majority are metastasis from a primary gonadal site (2). Primary extragonadal germ cell tumor is extremely rare. The rarity of primary extragonadal germ cell tumors, along with their nonspecific clinical presentation and imaging characteristics as well as their frequently mixed histological elements, presents a diagnostic and therapeutic challenge in clinical practice (3). Here, we report a large NSGCT arising from the right kidney in an adult male. Till date, to the best of our knowledge, there are only 30 cases reported worldwide of an adult primary renal germ cell tumor (4).

## Case Presentation

A 37-year-old gentleman presented with a complaint of right upper abdominal pain for 6 months, which was insidious in onset, constant, and mild in intensity. There was no history of hematuria, smoking, any comorbidities, or past surgery. He was normotensive. Upon abdominal examination, a 5 cm firm abdominal mass was palpated in the right upper quadrant and was associated with mild tenderness.

**Figure 1:**
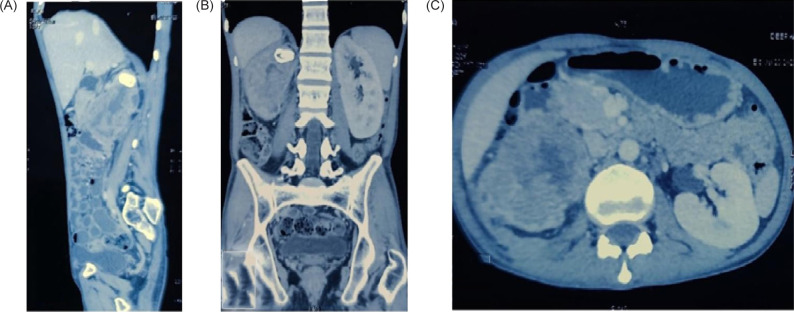
Sections of CECT abdomen (A) sagittal, (B) coronal, (C) axial. Suggestive of a deformed right kidney with soft tissue mass the size of 11 × 7 × 6.5 cm in the mid and lower pole extending to the hilar region with the compression of pelvicalyceal system and an upper pole calculi the size of 2.4 × 1.2 cm. Post contrast examination showed inhomogenous enhancement of the mass with no evidence of contrast excretion from the kidney.

**Figure 2:**
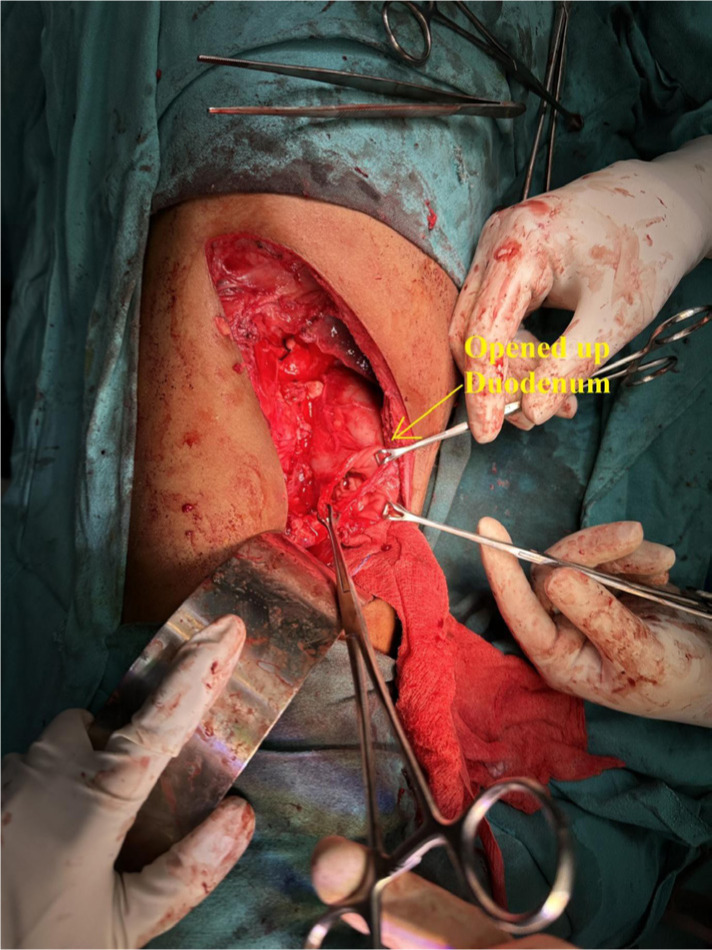
Intraoperative picture. Suggestive of opened up duodenum after radical nephrectomy.

**Figure 3:**
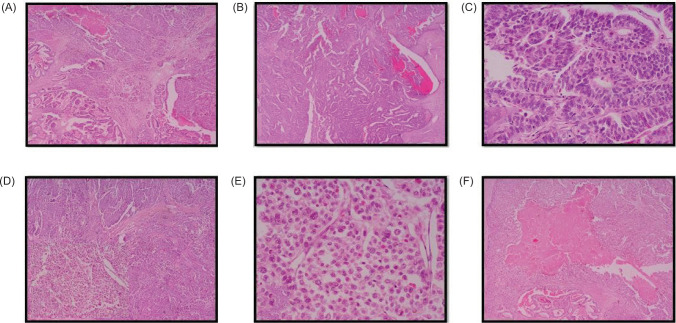
Microscopic images of the tumour. (A) Tumor with dual morphology H&E, 4x; (B) Tubulo-papillary architecture H&E, 4x; (C) Lined by columnar epithelial cells with nuclear pseudostratification with enlarged moderately pleomorphic oval to elongate nuclei, finely dispersed chromatin, visible nucleoli, and pale eosinophilic scant cytoplasm. H&E, 40x; (D) Tumor foci arranged in lobules, nests and sheets separated by intervening delicate fibrovascular septae H&E, 4x; (E) Large polygonal cells with moderate to markedly pleomorphic enlarged irregular nuclei, with dispersed chromatin, visible to prominent nucleoli and scant eosinophilic to clear cytoplasm H&E, 40x; (F) Intervening large areas of necrosis H&E, 4x.

**Figure 4:**
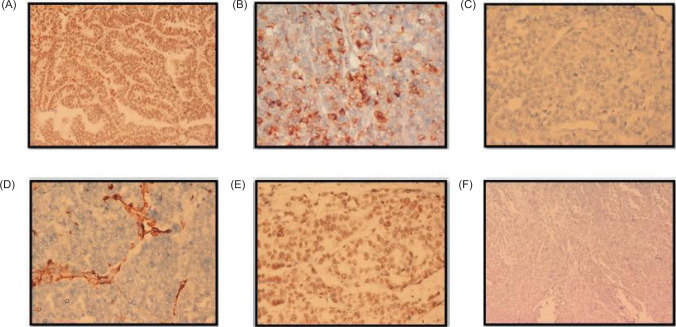
Immunohistochemistry images. (A) Near diffuse positivity in the tumor cells for OCT-3/4 of moderate to strong intensity, 20x; (B) Patchy cytoplasmic granular staining for AFP is noted mostly in the solid areas, 20x; (C) Negative for PAX-8, 40x; (D) Negative for Vimentin 40x; (E) Retained nuclear expression of INI-1 40x; (F) Negative for CD30, 10x.

**Figure 5:**
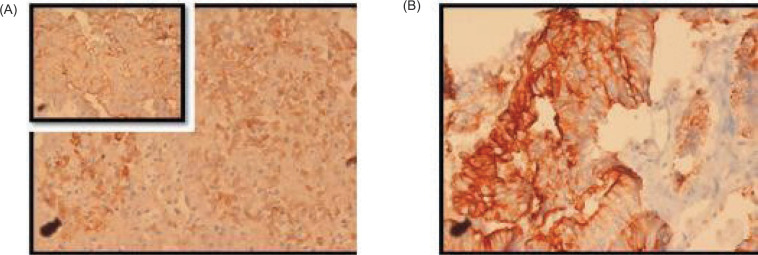
Immunohistochemistry. (A, B) Focal incomplete membranous staining for CK-7 and CK-20 is noted in the papillary areas, DAB, 40x.

Examination of both testes revealed normal findings. Abdominal and pelvic computed tomography (CT) scan revealed a deformed right kidney with soft tissue mass of size 11 × 7 × 6.5 cm in the mid and lower pole extending to the hilar region, with compression of pelvicalyceal system and an upper pole calculus of size 2.4 × 1.2 cm (Figure 1). Post contrast examination showed inhomogenous enhancement of the mass with no evidence of contrast excretion from the kidney. The CT of his chest showed no nodules or pulmonary metastases. The patient underwent a right open radical nephrectomy with lymph node dissection. Intraoperative, a 15 × 7 cm right renal mass was noted infiltrating the psoas muscle, duodenum, and liver. Additionally, a few enlarged retroperitoneal lymph nodes were also noted. The procedure involved the excision of the infiltrated mass in the duodenum with subsequent repair of the duodenum and the creation of feeding jejunostomy and retrograde duodenostomy (Figure 2). The histopathology report indicated a high-grade malignant NSGCT. Additionally, the submitted lymph nodes tested negative for malignancy. Immunohistochemistry performed showed diffuse positivity in the tumor cells for OCT-3/4 of moderate to strong intensity; patchy cytoplasmic granular staining for alpha-fetoprotein (AFP) was noted mostly in the solid areas—focal cytoplasmic granular positivity for alpha-methylacyl-CoA racemase (AMACR) and focal membranous positivity for CD10—and were negative for PAX-8, CD117, GATA-3, Vimentin, and CD30 (Figures 3 and 4). There was no loss of expression of INI-1; focal incomplete membranous staining for CK-7 and CK-20 was noted in the papillary areas (Figure 5).

The histopathology report indicated a high-grade malignant nonseminomatous germ cell neoplasm resembling in areas in morphology as a yolk sac tumor. The tumor cells showed a heterogeneous pattern of arrangement with a tubulopapillary architecture, noted in areas composed of columnar epithelial cells displaying nuclear pseudo stratification, enlarged moderately pleomorphic oval to elongate nuclei, visible nucleoli, and scant eosinophilic cytoplasm. At places, a solid pattern with large polygonal and pleomorphic cells with conspicuous mitotic activity was seen arranged in lobules, nests, and sheets. Intervening foci of necrosis was present.

Serum tumor markers were sent following the histopathological report. Serum AFP levels were raised with a value of 1550.43 ng/ml. Beta human chorionic gonadotropin (HCG) and lactate dehydrogenase (LDH) were normal. Ultrasonography of the testis was normal, ruling out testicular primary. Postoperative period was uneventful and the patient was discharged after 2 weeks. In view of the intraoperative duodenal infiltration, the adequacy of resection could not be guaranteed, and hence the patient underwent four cycles of adjuvant Bleomycin-Etoposide-Cisplatin (BEP) chemotherapy. Patient is fine at 6 months follow-up after chemotherapy.

## Discussion

Primary extragonadal germ cell tumors are rare, and only a few cases involving intrarenal tumors have been reported, particularly in the pediatric age group (4–8). It is exceptionally rare in adults. Only 30 case reports have been reported in adults of germ cell tumor originating primarily from the kidney (1, 9, 10). Complete surgical resection poses a significant challenge in such cases due to the involvement of major vessels and surrounding structures.

Given the rarity of these cases, there is limited literature available detailing effective therapies. Consequently, patients with primary germ cell tumors are typically managed similar to advanced stage or metastatic testicular germ cell tumor patients (11).

The current standard treatment involves a combination of BEP chemotherapy, which has demonstrated high efficacy against malignant germ cell tumors (12–17). However, the most effective management strategy appears to be surgical resection in combination with either neoadjuvant or adjuvant chemotherapy (8, 18, 19).

Due to the dearth of literature regarding management of such cases and following the favorable outcome of our patient after surgical resection and adjuvant chemotherapy, we find it necessary to report this case.

At initial presentation, this tumor was favored to be a renal cell carcinoma based on imaging characteristics; therefore, open radical nephrectomy was planned.

Due to intraoperative duodenal infiltration, the adequacy of resection could not be guaranteed, strengthening the indication for adjuvant chemotherapy.

The heterogeneous morphology constituting of papillary and solid patterns noted within the same tumor coupled with immunohistochemical positivity for AFP favored a diagnosis for a NSGCT with yolk sac tumor as the most likely possibility in this scenario. In this case, although OCT-3/4 positivity was noted, the lack of staining for CD117 and CD30 on immunohistochemistry and the raised serum AFP levels excluded the close differentials of seminoma and embryonal carcinoma.

A renal primary was excluded by the immunohistochemical negative staining for PAX 8 and Vimentin.

Following the histopathological confirmation of a NSGCT, the patient was assessed for a gonadal primary with ultrasound, demonstrating the absence of testicular tumors. Serum AFP levels were only measured following the confirmation of NSGCT on histopathology. Given the aggressive nature of such tumors, further studies are warranted to standardize investigative and management approaches aiming to enhance patient outcomes.

## Conclusion

The aim of this case report is to include NSGCT in the list of potential differential diagnoses for renal masses. Considering the aggressive nature of NSGCT, early diagnosis and management are essential for favorable outcomes. The combination of surgical resection and chemotherapy proves to be an effective treatment strategy for primary renal NSGCT.
